# Population Genetics, Demographic History, and Potential Distributions of the New Important Pests *Monolepta signata* (Coleoptera: Chrysomelidae) on Corn in China

**DOI:** 10.3390/insects16030323

**Published:** 2025-03-19

**Authors:** Yang Liu, Yacong Ge, Liming Wang, Jingao Dong, Zhenying Wang, Yuyu Wang

**Affiliations:** 1 College of Plant Protection, Hebei Agricultural University, Baoding 071001, China; yangliu010204@126.com (Y.L.); ayacong@163.com (Y.G.); wanglm1990@126.com (L.W.); 2 Institute of Plant Protection, Chinese Academy of Agricultural Sciences, Beijing 100193, China

**Keywords:** *Monolepta signata*, genetic diversity, phylogeography, potential suitability areas

## Abstract

*Monolepta signata* are polyphagous pest widely distributed in China, and the damage as well as economic losses it caused were increasing in recent years. Knowledge of species diversity, population structure and habitat suitability could enhance the efforts of pest control. Here, we sampled the populations of *M. signata* in almost all of China’s major corn-producing regions. A total of 568 sequences were obtained from each gene. There were 48, 29, and 30 haplotypes of *COI*, *ITS2* and *EF-1α*, respectively. The genetic distance between the HuangHuaiHai population and other populations was the largest. There were 61.90%, 71.43% and 61.90% of *Nm* values smaller than 1 in *COI*, *ITS2* and *EF-1α*, respectively, which indicated that gene flow between most populations was weak. The degree of differentiation in most populations of *M. signata* was relatively high. The population of *M. signata* has also experienced rapid expansion. Population history dynamic analysis showed that the effective population size of *M. signata* remained relatively stable before 0.075 Ma. There was a slow contraction trend from 0.075 to 0.010 Ma. It has been rapidly and continuously expanding since 0.010 Ma. Among the investigated geographical populations, the “yellow-spot type” was only present in the populations of southern and southwestern regions, while the “two-spot type” and “four-spot type” were widely distributed in all other geographical populations. Predictions of the potential distribution areas of *M. signata* indicated that the northeast and north China regions will remain being the high suitability areas of *M. signata* in the future. Our results will not only facilitate studies on the phylogeography of *M. signata* but also benefit the effective monitoring and management of this agricultural pest.

## Simple Summary

The adults of *Monolepta signata* mainly feed on corn silks, anther and kernels at the filling stage, which result in great loss of yield and quality. Herein, we studied the population genetics and demographic history of *M. signata* based on mitochondrial genes (*COI*) and nuclear genes (*ITS2*, *EF-1α*). Based on the data of environmental factors and known sample points, the fitness of *M. signata* was also analyzed according to mathematical statistics and ecological niche theory. The distribution of the suitable habitat of *M. signata* in China and center-of-mass transfer under different climatic scenarios in the present and future time periods were predicted. The areas of potential serious damage in the future were also predicted. The population of *M. signata* has also experienced rapid expansion. The damage caused by *M. signata* in Xinjiang will be worse and more attention should be paid on.

## 1. Introduction

*Monolepta signata* (Coleoptera, Chrysomelidae) (Oliver 1808) are polyphagous pest widely distributed in China, whose hosts include ferns, dicotyledons and monocotyledons, with a total of 218 species (including subspecies and varieties) in 45 families of three classes [1]. *Monolepta signata* has a long damage period and strong adaptability to high temperatures [2]. Adults mainly feed on corn silks, anthers and kernels during the grain-filling stage, causing significant yield and quality losses. Feeding damage also facilitates fungal infections (e.g., *Fusarium* spp.), leading to corn ear rot [3,4,5,6,7,8]. *M. signata* damages cotton bracts and corollas, resulting in incomplete corolla and stamen exposure, which could affect cotton production and quality, particularly under heavy infestation [9]. *M. signata* can also infest vegetable leaves, meristems, and other plant parts [10]. *M. signata* was not only distributed in China, Russia (Siberia), Korea, Japan [11] but also in Bangladesh, Nepal and India [12,13,14,15,16,17]. The original distribution area of *M. signata* includes East Asia and Southeast Asia, specifically in regions such as Russia (Siberia), China, South Korea, Japan, the Philippines, Indonesia, Singapore, Malaysia, northern Vietnam, Myanmar, and eastern India [18,19]. Over time, the distribution range of *M. signata* has significantly expanded, spreading across nearly 30 provinces, autonomous regions, and municipalities in China, with particular severe damage reported in northern regions of China [20,21].

Due to changes in climate and cultivation practices, the reports of damage caused by *M. signata* have been increasing since 2000. In 2008, *M. signata* caused severe damage in Chencang District, Baoji City, Shaanxi Province, affecting 75.3% of the farmland, with the infestation rate reaching 100% in summer cornfields [22]. The corn silks were completely gnawed off, resulting in a yield loss of approximately 15% in the damaged fields in the most severely affected areas [22]. In 2010, cornfields in Qiqihar City, Heilongjiang Province, were severely damaged by *M. signata*, with the total affected area across the city reaching 303,000 hectares [23]. In 2014, large-scale damage caused by *M. signata* occurred in Xinxiang County, Henan Province, where 70% of the farmland was affected and the damaged plant rate reached 27% [24]. The occurrence regions of this pest have also expanded, resulting in an escalating economic loss of many crops such as soybeans, corn, peanuts, cotton, millet, and vegetables [25,26]. In recent years, the occurrence acreage of *M. signata* on corn has been increasing in China. *M. signata* has become an important pest on spring corn in north China and irrigated corn in northwest China as well as summer corn in the HuangHuaiHai region [27]. In particular, the northern part of China has suffered severe damage. Currently, most of the studies on *M. signata* are focused on the pattern of occurrence and effective control [3,4,28,29,30]. However, there are few studies on population genetics and demographic history of *M. signata* [31,32,33].

Phylogeography is an integrative field of science linking micro and macro evolutionary processes, contributing to the inference of vicariance, dispersal, speciation, and other population-level processes [18]. Phylogeography can provide strong support for addressing the relationships between current distribution patterns of species and climate change, geographic isolation, as well as clarify the timing of divergence of clades, origins and diffusion paths of species [21,34,35,36]. The maximum entropy model (MaxEnt) [37] is based on the maximum entropy theory, with species distribution data and climate variables as the foundation, and simulates the potential geographical distribution range of species through mathematical models [10]. The study of genetic diversity and population dynamics of pest populations not only clarifies scientific issues such as pest occurrence, gene exchange, genetic evolution and pesticide resistance, but also provides a scientific basis for optimizing ecological management and developing integrated pest management (IPM) [38,39].

Molecular markers, due to their advantages such as the independence from gene expression status, high reproducibility, and excellent stability, have been extensively utilized in multiple disciplines, including genetic structure analysis, assessments of population genetic diversity, species identification, phylogeography, and phylogenetic relationship investigations [40,41,42,43]. Among various molecular markers, mitochondrial DNA (mtDNA) is widely used in phylogenetic studies due to its structural simplicity, ease of amplification, and higher evolutionary rate, which enhances resolution in closely related species [44,45,46,47]. Nuclear gene sequences are widely used in phylogenetic reconstruction, gene loss analysis, population genetic structure, and biogeographic studies [48]. The Internal Transcribed Spacer (*ITS*) regions within nuclear rDNA are characterized by low selection pressure and rapid evolutionary rates, making them suitable for identifying closely related insect species, reconstructing phylogenetic relationships, and conducting population-level studies [49,50]. Notably, *ITS2* has been widely applied in species typing [51,52] and phylogeographic investigations [53]. The eukaryotic elongation factor *EF-1α* plays a critical role in protein translation [54]. Numerous studies have employed *EF-1α* in conjunction with mitochondrial genes to explore insect genetic diversity and phylogeographic patterns [55,56]. The combination of mitochondrial and nuclear genes, which exhibit distinct inheritance patterns and evolutionary rates, offers a more reliable approach for investigating population genetic structure and inferring population dynamics [57].

Herein, we studied the population genetics and demographic history of *M. signata* based on mitochondrial genes (*COI*) and nuclear genes (*ITS2*, *EF-1α*). Based on the data of environmental factors and known sample points, the suitability areas of *M. signata* were also analyzed according to mathematical statistics and ecological niche modeling. The distribution of suitability areas of *M. signata* in China and geographic distribution shifts under different climatic scenarios in the present and future time periods were predicted. The areas of potential serious damage in the future were also predicted.

## 2. Materials and Methods

### 2.1. Sample Collection

**Figure 1:**
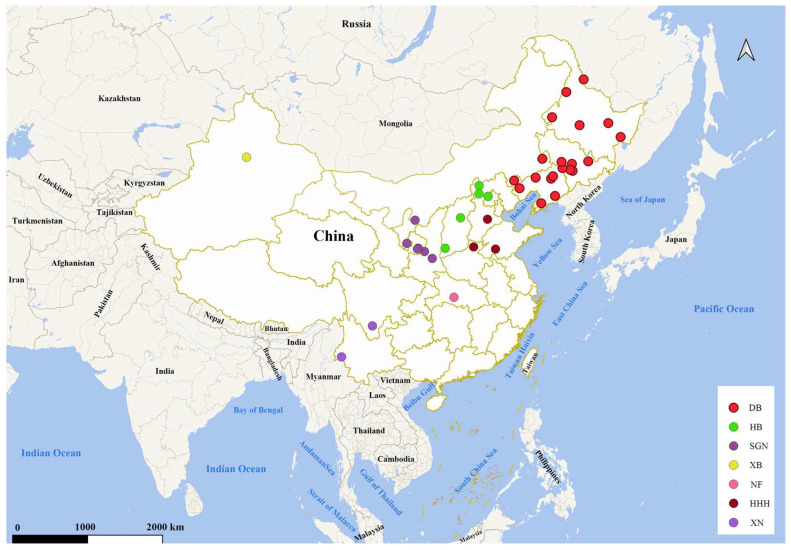
The geographic coordinates of 38 collection sites. Map approval number: GS(2024)0650.

The specimens used in this study were collected from Heilongjiang, Jilin, Liaoning, Inner Mongolia, Xinjiang, Hebei, Shaanxi, Yunnan, Sichuan, etc. (Table S1). A total of 568 specimens of *M. signata* from 38 localities were collected, and their coordinates were plotted using QGIS v.3.28 [58] (Figure 1). The administrative division map of China was downloaded from the National Geomatics Center of China (http://ngcc.cn/, accessed on 25 February 2025). Adult specimens of *M. signata* were sampled during 2020 and 2021 from almost all major corn planting areas in China, to comprehensively represent the current distribution of *M. signata*. The *M. occifluvis* specimens collected in 2018 were used as the outgroup for phylogenetic analyses (Table S1). All specimens were preserved in 95% ethanol and stored at −20 °C at Hebei Agricultural University, Baoding, China.

### 2.2. DNA Extraction and Sequencing

Genomic DNA was extracted from the thoracic muscle tissue using the Rapid Genomic DNA Kit (Biomed Biological Technology Co., Ltd., Beijing, China) following the instructions. The DNA concentration was measured using a nucleic acid protein analyzer (Thermo Scientific, Waltham, MA, USA) and preserved at −20 °C. The PCR primers used in this study were synthesized by Biomed (Beijing, China). *COI* and *ITS2* were amplified using the 25 μL system: 2× Taq PCR Mix 12.5 μL, Forward primer 1 μL, Reverse primer 1 μL, template DNA 1 μL, and ddH_2_O 9.5 μL. The PCR conditions were: initial denaturation at 95 °C for 30 s; 40 cycles of denaturation at 95 °C for 10 s, annealing (*COI*: 48 °C, *ITS2*: 58 °C) for 50 s, and extension at 65 °C for 1 min; followed by a final extension at 65 °C for 10 min. The barcoding region of *COI* was amplified using the primers S-jerry (5′-CAACATYTATTYTGATTYTT-3′) and S-pat (5′-GCACTAWTCTGCCATATTAGA-3′) [59]. And *ITS2* was amplified using the primers ITS-J-5.8S (5′-TGGRTCGATGGAGAACGCAGC-3′) and ITS2-N-610 (5′-TCTCACCTGCTCTGAGGTCGATAT-3′) [60]. The nested PCR procedure of *EF-1α* gene fragment was conducted with reference to two pairs of primers, i.e., EFS149 (5′-GARAARGARGCNCARGARATGGG-3′) and EFA1106 (5′-GTATATCCATTGGAAATTTGACCNGGRTGRTT-3′), EF1a-SN (5′-TGGGAAAAGGYYCCTTCAAATATGC-3′) and EF1a-AN (5′-CRTRACCACGACGYAATTCTTTGACAG-3′) [61]. PCR products were examined using 1% agarose gels with ethidium bromide following electrophoresis and sent to Biomed (Beijing, China) for sequencing in both directions.

Bidirectional sequencing reads were assembled in Cexpress [62]. *COI* and *EF-1α* sequences were validated by translating to amino acids in DNAMAN v.6.0 (Lynnon Biosoft, San Ramon, CA, USA) and then blasting with sequences on NCBI (https://www.ncbi.nlm.nih.gov/, accessed 6 May 2024) while the *ITS2* sequences were verified by blasting with sequences on NCBI directly.

### 2.3. Genetic Diversity and Historical Dynamics Analysis

Single-nucleotide polymorphism (SNPs) and parsimony-information-sites were calculated using MEGA v7.0 [63]. The extent of haplotype diversity (Hd) and nucleotide diversity (π) of each population were calculated using DnaSP v5.0 [64]. The haplotype network was constructed using PopART v1.7 [65] based on the median-joining algorithm. The genetic differentiation indices and analysis of molecular variance (AMOVA) of different *M. signata* geographical populations were analyzed by Arlequin v3.5 [66]. The genetic distances within and among populations were calculated using MEGA v7.0 [63] based on the Kimura 2-parameter genetic distance model.

Mismatch distribution analysis was conducted in DnaSP v5.0 [64] to test whether the population of *M. signata* had undergone recent demographic expansion. Tajima’s *D* [67] and Fu’s *Fs* [68] neutrality tests were performed using Arlequin v.3.5 [66]. The Bayesian Skyline Plot (BSP) was plotted using BEAST v1.8 [69] based on *COI* gene under the GTR + G model. A Coleoptera-specific *COI* substitution rate of 1.77% per million years [70] was applied to calibrate the molecular clock in BEAST v1.8 [69]. The BSP was visualized in Tracer v1.4 [71] using the posterior tree distribution from BEAST v1.8 [69].

### 2.4. Phylogenetic Analysis

Phylogenetic trees were constructed based on *COI*, *ITS2* and *EF-1α* genes of *M. signata* based on maximum likelihood (ML) and Bayesian inference (BI) methods using PhyloSuite v1.16 [72] with *M. occifluvis* as the outgroup. The best models of ML analyses were selected by ModelFinder [73], i.e., GTR + T + F for *COI*, HKY + G + F for *ITS2* and K80 + I for *EF-1α*. ML trees were inferred using IQ-TREE v.1.6.10 [74] with 1000 ultrafast bootstraps. BI analyses were conducted using MrBayes v.3.2.2 [75] under the best substitution models selected by ModelFinder [73], i.e., TIM2 + I + F for *COI*, HKY + G4 + F for *ITS2* and TNe + R2 for *EF-1α*. Bayesian Markov chain Monte Carlo (MCMC) simulations were run for 1 × 10^8^ generations, sampling every 5000 generations and stopped when the average standard deviation of split frequencies < 0.01. The first 25% trees were discarded as burn-in. Finally, the resulting trees were visualized by Figtree v.1.4.1 [76].

### 2.5. Divergence Time Analysis

Molecular calibration of evolutionary rates for *COI* gene in Coleoptera (1.77% bases per million years) [70] was used to estimate the divergence time since lack of appropriate fossil records. The divergence times among *M. signata* haplotypes and its geographic populations were estimated using BEAST v1.8 [69] under the GTR + G model with strict molecular clock. Two independent MCMC runs were performed for 1 × 10^8^ generations with sampling at every 5000 generations. The first 10% trees were removed as burn-in. Finally, the tree was visualized by Figtree v1.4.1 [74].

### 2.6. Potential Distribution of M. signata

There were 144 distribution points of *M. signata* in China used in this study, including field collection points and data points according to related literature and the Global Biodiversity Information Facility (GBIF) database.

To reduce the bias in the amount of occurrence data on the area, the localities were selected using ENMtool 1.3 [77], keeping only one average data for multiple distribution points located within the same raster (~1.5 km × ~1.5 km) (Table S3). There were 19 bioclimatic variables (BIO) in WorldClim (https://www.worldclim.org/data/v1.4/worldclim14.html, accessed on 15 May 2024) cited to model the distribution of *M. signata*. Pearson correlation analysis of the 19 variables was performed using R package v.3.4.2 [78], selecting variable factors with correlation coefficients |R| < 0.8. The spatial resolutions used for climate variable data were 2.5 arc-minutes. In order to investigate the changes in the distribution pattern and range of *M. signata* under different climate scenarios, the latest Coupled Model Intercomparison Project Phase 6 (CMIP6) Shared Socioeconomic Pathways (SSP) data were used in this study. Two climate scenarios (SSP126 and SSP585) for the present (1970–2000) and future (2041–2060 and 2061–2080) were selected.

The suitable geographic distribution of *M. signata* in China was analyzed using MaxEnt v 3.4.1 [79]. The maximum training sensitivity plus specificity logistic threshold (MTSS) was considered to be the most accurate method that predicted the presence or absence of species. The average of 10 replicates of the MTSS was used as a reference. The suitability zone of *M. signata* was classified into four classes according to the Natural Breaks in ArcToolbox of ArcGIS [80], with 0 ≤ *p* < 0.15 (non-suitability zone), 0.15 ≤ *p* < 0.3 (low suitable zone), 0.3 ≤ *p* < 0.5 (medium suitable zone), and 0.5 ≤ *p* ≤ 1 (high suitable zone) [81]. The spatial changes in overall suitable habitats were analyzed through the changes in centroid position at different periods of *M. signata*, taking their suitability areas as a whole. The direction and distance changes in fitness zone of *M. signata* were analyzed using the SDMtoolbox of ArcGIS [80].

## 3. Results

### 3.1. Genetic Structure

According to the geographical distribution, all samples were divided into seven geographical populations, namely DongBei population (DB), HuaBei population (HB), HuangHuaiHai population (HHH), ShanGanNing population (SGN), XiBei population (XB), NanFang population (NF) and XiNan population (XN).

Comparative analysis of mitochondrial (*COI*) and nuclear (*ITS2*, *EF-1α*) markers revealed distinct diversity patterns (Table 1). Geographic heterogeneity was evident in the genetic diversity patterns of the studied populations. The XB population exhibited the lowest diversity values (*COI*: *π* = 0.003; *ITS2*: *π* = 0.002), suggesting long-term isolation likely driven by the arid environmental conditions and geographic barriers in China’s northwestern regions. In contrast, the SGN population displayed a nuclear-specific reduction in diversity (*EF-1α*: *π* = 0.0008). The mitochondrial *COI* exhibited the highest haplotype diversity (Hd = 0.526) and nucleotide diversity (π = 0.006), consistent with its rapid evolutionary rate and maternal inheritance mode, whereas nuclear markers showed reduced variation (*ITS2*: Hd = 0.433, π = 0.004; *EF-1α*: Hd = 0.472, π = 0.002). Notably, haplotype sharing was limited across populations, with only 15% (7/48) of *COI* haplotypes distributed in multiple regions, compared to 24–33% shared haplotypes in nuclear loci (7/29 for *ITS2*; 10/30 for *EF-1α*), suggesting a stronger phylogeographic structure in the mitochondrial genome (Table S2).

### 3.2. Phylogenetic Analyses

**Figure 2:**
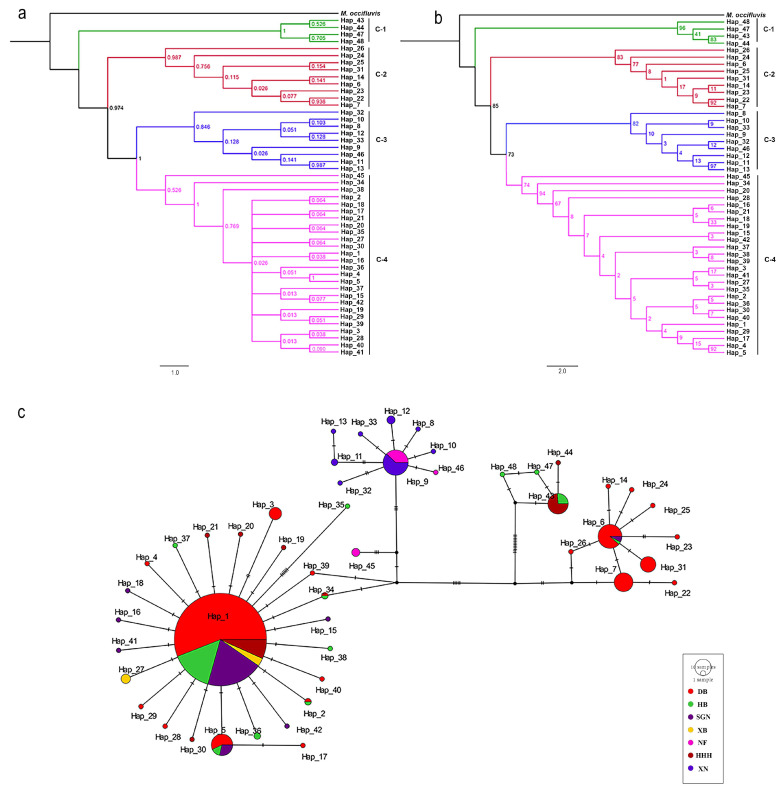
Phylogenetic analysis and haplotype network of *Monolepta signata* based on the *COI* sequences. (**a**) Bayesian phylogenetic tree. (**b**) Maximum likelihood tree. (**c**) Haplotype network. Haplotypes are color-coded according to the population. Bright red represents the DB population. Green represents the HB population. Dark purple represents the SGN population. Yellow represents the XB population. Pink represents the NF population. Dark red represents the HHH population, and dark blue represents the XN population.

**Figure 3:**
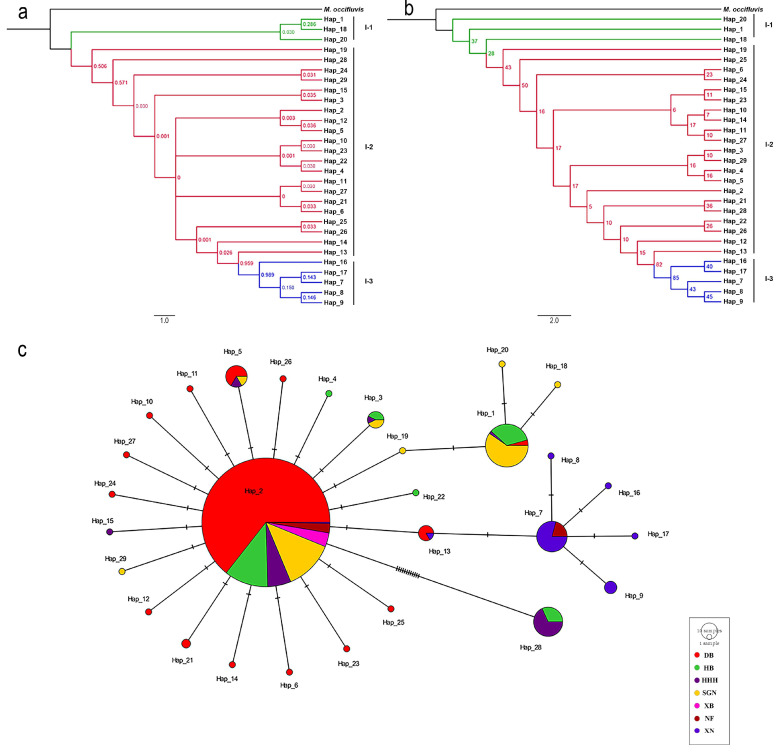
Phylogenetic analysis and haplotype network of *Monolepta signata* based on the *ITS2* sequences. (**a**) Bayesian phylogenetic tree. (**b**) Maximum likelihood tree. (**c**) Haplotype network. Haplotypes are color-coded according to the population. Bright red represents the DB population. Green represents the HB population. Dark purple represents the HHH population. Yellow represents the SGN population. Pink represents the XB population. Dark red represents the NF population, and dark blue represents the XN population.

**Figure 4:**
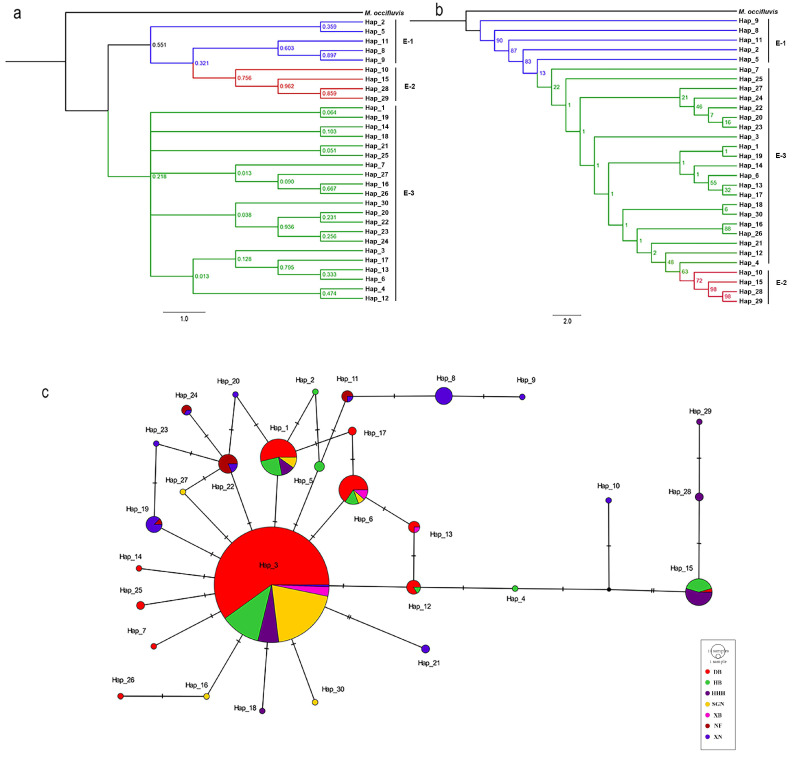
Phylogenetic analysis and haplotype network of *Monolepta signata* based on the *EF-1α* sequences. (**a**) Bayesian phylogenetic tree. (**b**) Maximum likelihood tree. (**c**) Haplotype network. Haplotypes are color-coded according to the population. Bright red represents the DB population. Green represents the HB population. Dark purple represents the HHH population. Yellow represents the SGN population. Pink represents the XB population. Dark red represents the NF population, and dark blue represents the XN population.

The haplotype distributions of the three genes are summarized in Table S2, with *COI*, *ITS2*, and *EF-1α* comprising 48, 29, and 30 haplotypes, respectively. Phylogenetic reconstructions based on Bayesian inference (BI) and maximum likelihood (ML) methods yielded congruent topologies for each gene (Figure 2, Figure 3 and Figure 4). Median-joining networks further corroborated these phylogenetic relationships, revealing distinct clustering patterns among markers with *COI* resolved four major clades, whereas *ITS2* and *EF-1α* grouped samples into three clades, likely reflecting differences in evolutionary rates and inheritance modes. Dominant haplotypes varied across markers. For *COI*, Hap_1 was the most frequent haplotype, predominantly distributed in northern populations (DB, HB, HHH, SGN, XB). For *ITS2*, Hap_2 was the most widespread, occurring across all seven geographic groups. For *EF-1α*, Hap_2 also dominated, presenting in six populations (DB, HB, HHH, SGN, XB, XN). Based on the phylogenetic tree topology and haplotype network structure, mitochondrial genes supported the division of *M. signata* from different regions of China into four distinct clades, whereas nuclear genes supported three major lineages.

### 3.3. Population Genetic Analysis

There were significant differences between the HHH population and the other six populations based on the pairwise differentiation coefficient (Table S6). The HHH population showed significant genetic differentiation compared to the DB, SGN, NF and XN populations (*Fst* > 0.25, *p* < 0.05) based on *COI*, to DB, SGN and XN populations (*Fst* > 0.25, *p* < 0.05) based on *ITS2*, and to DB, SGN and NF populations (*Fst* > 0.25, *p* < 0.05) based on *EF-1α*. There are 61.90%, 80.95% and 71.43% of *Fst* values exhibit high genetic differentiation (*Fst* > 0.15) in *COI*, *ITS2* and *EF-1α*, respectively. Analysis of gene flow among different populations showed that there are 61.90%, 71.43% and 61.90% of *Nm* values smaller than 1 in *COI*, *ITS2* and *EF-1α*, respectively, indicating that the gene flow of most populations were weak. Results from AMOVA analysis revealed that the main source of genetic variation existed within populations, whereas the remaining came from variation among populations for all these three genes (Table 2).

### 3.4. Divergence Time and Historical Demographic Reconstruction

**Figure 5:**
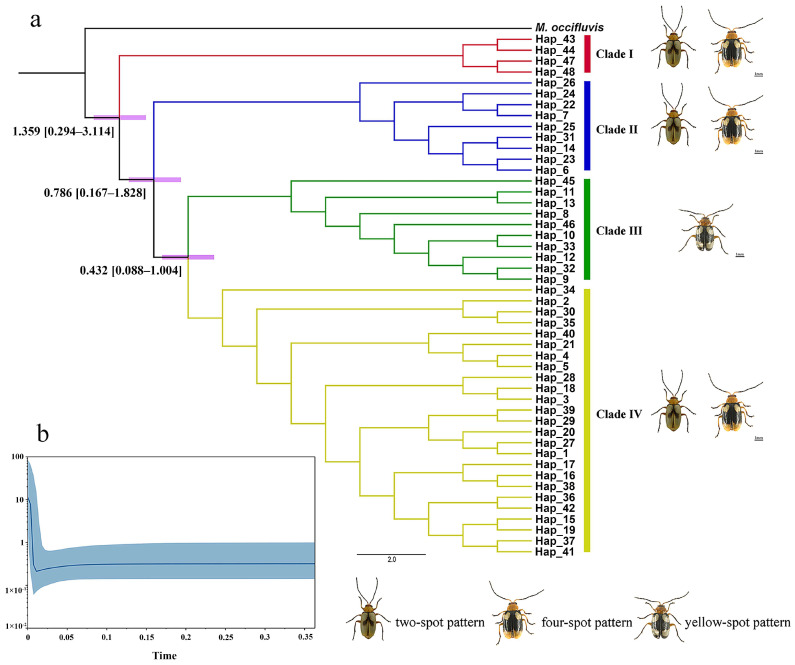
Divergence time estimation and Bayesian skyline analysis for *Monolepta signata* using *COI* gene. (**a**) Divergence time estimation. Node mean age estimates are provided with their respective 95% highest posterior density (HPD) intervals, denoted by purple bars. (**b**) Bayesian skyline analysis. The abscissa represents time. Unit: million years. The ordinate represents effective population size.

The divergence time was estimated based on the phylogeny of the *COI* gene (Figure 5a). The initial divergence with extant *M. signata* in China was estimated to occur at 1.359 Ma (95% HPD = 0.294–3.114 Ma). *M. signata* further separated into Clade I at 1.359 Ma (95% HPD = 0.294–3.114 Ma), which comprised the HB and HHH populations, exhibiting both “two-spot type” and “four-spot type” patterns. Clade II, which also included “two-spot type” and “four-spot type” patterns, including DB, SGN, and HB populations, diverged from Clade III + Clade IV at 0.786 Ma (95% HPD = 0.167–1.828 Ma). Clade III, which included NF and XN populations with “yellow-spot type” patterns, diverged from Clade IV at 0.432 Ma (95% HPD = 0.088–1.004 Ma). Clade IV, including DB, HB, SGN, XB, and HHH populations, had both “two-spot type” and “four-spot type” patterns.

**Figure 6:**
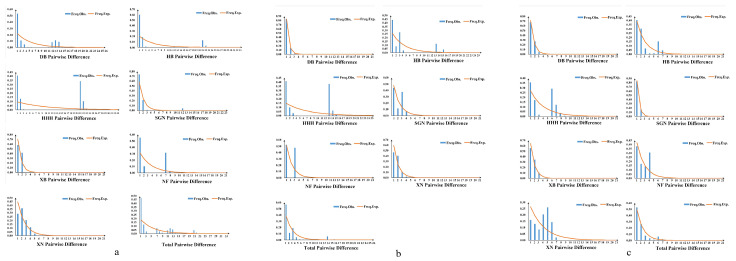
Mismatch distribution based on data from geographic population for *COI* (**a**), *ITS2* (**b**) and *EF-1α* (**c**) genes of *Monolepta signata*.

Tajima’s *D* and Fu’s *Fs* values of all populations for these three genes were significantly negative (*p* < 0.05), indicating recent population expansion occurred in *M. signata* (Table 3). The BSP results showed that the population of *M. signata* may have experienced three main demographic history periods (Figure 5b). The effective population size of *M. signata* remained relatively stable before approximately 0.075 Ma. There was a slow contraction trend between 0.075 and 0.010 Ma. The effective population size of *M. signata* has been experiencing rapid and sustained expansion from 0.010 Ma until now. Meanwhile, the mismatch distribution analysis based on data from geographic populations of *M. signata* did not display a single-peak curve, indicating that the population has not experienced recent expansion, which is conflicted with the results of the neutrality tests (Figure 6). We have also conducted mismatch distribution analysis using data from each genetically divergent clade instead of data from geographic populations, which is consistent with the results of geographic population (Figure S1).

### 3.5. Potential Distribution Prediction

A relatively high AUC value was obtained from the current potential distribution (AUC =  0.984), indicating good predictive model performance (Figure S2). Correlation analysis and model contribution screening of 19 climate variables were shown in Figure S2, with nine climate variables with high adaptability selected in this study. The contribution rate of environmental variables to the distribution of *M. signata* was shown in Table S7.

The climate variables that contributed more than 10% to the prediction of the MaxEnt model were bio18, bio4, and bio10, while the cumulative contribution of the three variables accounted for 66.8%. The replacement significance values that contributed more than 10% were bio4, bio1 and bio5, while the cumulative replacement significance values of the three climate variables accounted for 87.9%. The Jackknife test of the regularized training gains for climate variables in MaxEnt demonstrated that four environmental variable factors, i.e., bio1, bio10, bio18, and bio5, have relatively large gains when used alone (Figure S3). Five variables (bio1, bio4, bio5, bio10 and bio18) dominated the distribution of *M. signata* in China. The response curve of main environmental variables was shown in Figure S4. The optimal mean annual temperature of *M. signata* was 5 °C. The optimum value for the variance in temperature change was 1500. The optimal maximum temperature in the hottest month was 28 °C. The optimum average temperature during the warmest season was 22 °C. In the warmest season, the occurrence rate of *M. signata* increases with the increase in precipitation until the precipitation reaches 2500 mm.

**Figure 7:**
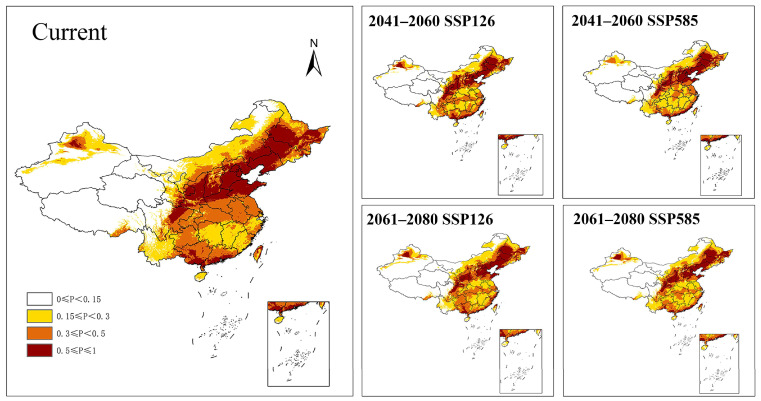
Modeled suitability areas of *Monolepta signata* under the current climate conditions and two future climate change scenarios (SSP126 and SSP585) in 2041–2060 and 2061–2080.

The prediction of the suitability areas of *M. signata* in China under three different periods is shown in Figure 7. In the current period (1970–2000), Qinghai Province and Xizang Autonomous Region are areas where *M. signata* is distributed sporadically, while other regions show potential distribution areas. The current optimal habitat of *M. signata* was mainly concentrated in the Northeast Plain, North China Plain, Middle and Lower Yangtze River Plain, Sichuan Basin, Loess Plateau and Junggar Basin. The high-suitability area of *M. signata* in China accounted for 15.52%, while the medium-suitability area accounted for 20.42% and the low-suitability area accounted for 20.10%, respectively (Table 4).

The low- suitability areas of *M. signata* in China under the future climate scenarios SSP126 and SSP585 increased, while the medium-suitability and high-suitability areas decreased, compared to the results of the distribution projections under current climate scenarios (Table 4). The pattern of suitability areas was more variable in the south-central region. There was a gradual shift from high and medium to low-suitability areas especially in Shandong, Henan, Jiangsu, Anhui, Hubei, Hunan, Jiangxi and Fujian Province (Figure 7).

**Figure 8:**
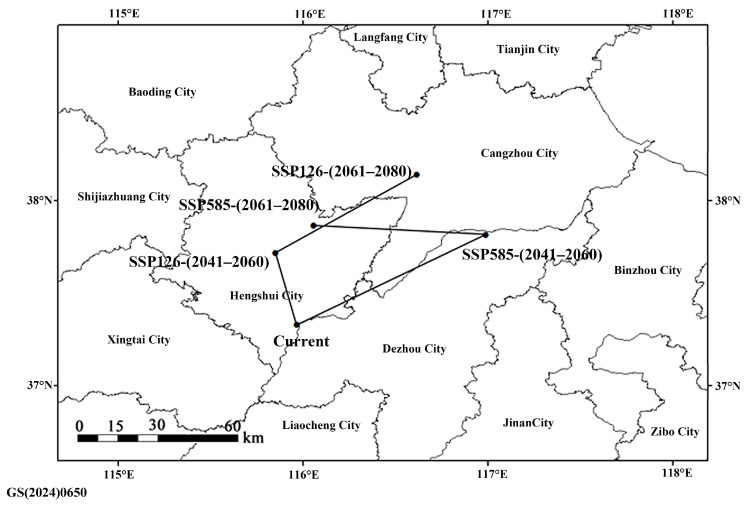
Centroid transfer in the suitability areas of *Monolepta signata* under different climate scenarios.

The center of mass of current suitability areas of *M. signata* was located in Gucheng County, Hengshui City, Hebei Province. From the current to the future (2041–2060 and 2061–2080) periods, the center of gravity distribution will still be in north China. The direction and distance of the center of gravity changed slightly under different climatic conditions (Figure 8). Under the SSP126 scenario, the centroid of the suitability areas shifted slightly to the northwest first and then to the northeast. Under the SSP585 scenario, its centroid tended to shift to the northeast first and then to the northwest. Further, the habitats of *M. signata* will all shift to the north in the future, indicating that the harm of *M. signata* will increase in the northern part of China in the future.

## 4. Discussion

### 4.1. Mitochondrial–Nuclear Discordance in Phylogenetic Resolution of M. signata

In the phylogenetic results, mitochondrial genes supported the division of *M. signata* from different regions of China into four distinct clades, whereas nuclear genes resolved three major lineages. The observed mitochondrial–nuclear discordance aligns with theoretical expectations, i.e., mitochondrial genes, due to their haploid and uniparental inheritance, have a smaller effective population size (effective population size ≈ 1/4 of nuclear genes), leading to faster lineage sorting and higher resolution of recent divergence events [82]. In contrast, nuclear genes, with their larger effective population size and biparental inheritance, are more likely to retain ancestral polymorphisms, obscuring finer phylogenetic signals [83].

### 4.2. Phenotypic Variation and Phylogeographic Patterns

The distribution of “two-spot type” and “four-spot type” across clades I, II, and IV, contrasted with the exclusive presence of the “yellow-spot type” in clade III, suggests a strong association between phenotypic traits and geographic regions (Figure 5a). This pattern likely results from continuous selection pressures along environmental gradients, leading to gradual phenotypic divergence and the formation of distinct ecotypes [84]. The geographic separation of the “four-spot type” and “yellow-spot type” populations, coupled with their genetic differentiation, may indicate ongoing speciation driven by local adaptation [85]. Future studies should focus on validating these patterns through offspring analysis and exploring the genetic basis of phenotypic variation.

### 4.3. Mitochondrial–Nuclear Discordance and Demographic History

The higher haplotype diversity (*Hd* > 0.5) and nucleotide diversity (*π* > 0.005) observed in *COI*, compared to the lower values in *ITS2* and *EF-1α* (*Hd* < 0.5, *π* < 0.005), reflect the faster evolutionary rate and smaller effective population size of mitochondrial DNA [86]. The relatively low nucleotide diversity in nuclear genes, despite moderate haplotype diversity, may indicate historical bottlenecks followed by rapid population expansion [87,88]. During bottlenecks, mitochondrial diversity is more severely reduced due to its haploid nature, whereas nuclear genes retain higher haplotype diversity through recombination [89,90]. Post-bottleneck, mitochondrial diversity recovers more slowly, while nuclear genes rapidly accumulate new haplotypes through mutation and recombination [91]. The observed patterns are consistent with rapid population expansion, during which mitochondrial diversity increased due to its higher mutation rate, while nuclear diversity remained constrained by recombination [92].

### 4.4. Genetic Differentiation and Gene Flow Barriers

The genetic differentiation analysis based on *COI*, *ITS2* and *EF-1α* showed significant differences between the HHH population and the other six populations, and significant genetic differentiation with some populations (*Fst* > 0.25, *p* < 0.5). The HHH population had a large genetic distance from other geographical populations. There was a negative correlation between gene flow and the genetic differentiation coefficient. The HHH population may have less gene exchange with other populations due to geographical barriers or other reasons, resulting in significant differences in the genetic differentiation coefficient between populations. Geographical isolation is considered an important factor affecting the genetic structure of species [93]. The HuangHuaiHai region is surrounded by mountains on three sides and faces the sea on one side. Coupled with the weak flight ability of *M. signata* [86], the obstruction of mountains such as the Qinling Mountains and the Taihang Mountains might be the main reasons for the large genetic distance and significant differentiation between the HHH population and other populations in China [94]. In contrast, gene flow between Chinese populations and those in Japan and North Korea may result from the dispersal of adult individuals by air currents. It is necessary to collect additional samples from countries and regions surrounding China to explore the formation causes of the current distribution pattern of *M. signata*.

### 4.5. Human Impacts and Host-Associated Differentiation

The lack of clear phylogeographic structure in China, as evidenced by the widespread distribution of haplotypes in the HB population, may reflect human-mediated dispersal through agricultural activities. Similar patterns have been observed in other pests, such as *Sitobion avenae*, where host crops significantly influence genetic structure [95]. Future studies should expand sampling to include more natural populations and different host crops to better understand the role of host-associated selection in shaping genetic diversity.

### 4.6. Environmental Constraints and Future Distribution

Temperature and precipitation were identified as the dominant factors limiting the distribution of *M. signata*, consistent with its known ecological requirements [96,97,98]. Under future climate scenarios (SSP126 and SSP585), northern regions (northeast China, north China, and Xinjiang) are projected to remain highly suitable, likely due to the expansion of corn and cotton cultivation, which are primary host crops [99,100,101,102]. In contrast, central and southern regions may experience reduced suitability, with high and medium suitable areas transitioning to low suitability. The northward shift of the centroid of *M. signata*’s distribution suggests increasing pest pressure in northern agricultural zones, necessitating targeted monitoring and control strategies.

## 5. Conclusions

This study provides a comprehensive analysis of the genetic diversity, population historical dynamics, and potential distribution of *M. signata*. Key findings reveal high genetic differentiation among populations, with larger genetic distances based on *COI* and *ITS2* compared to *EF-1α*, indicating marker-specific evolutionary patterns driven by differences in effective population sizes and mutation rates. The primary source of genetic differentiation stems from significant intraspecific variation within populations, highlighting the role of local adaptation and geographic isolation in shaping population structure. Future distribution predictions suggest that northeast China, north China, and Xinjiang will remain highly suitable for *M. signata*, with increasing pest pressure in northern agricultural zones due to the expansion of corn and cotton cultivation. In central and southern China, shifts in suitability patterns are evident, with areas transitioning from low to medium suitability likely emerging as new hotspots for pest outbreaks, necessitating proactive monitoring and control measures.

Our findings have important implications for pest management. The genetic structure of *M. signata* provides a foundation for understanding its evolutionary history and adaptive potential, while the predicted distribution patterns offer critical insights for developing targeted monitoring and control strategies. Future research should focus on expanding sampling to include more natural populations and additional host crops, investigating the genetic basis of local adaptation and host-associated differentiation, and integrating genomic data to refine predictions of *M. signata*’s response to climate change.

## Figures and Tables

**Table 1:** Haplotype distribution and genetic diversity of *Monolepta signata* based on *COI*, *ITS2* and *EF-1α* genes.

Gene Name	Population Code	Haplotype Diversity (Hd)	Nucleotide Diversity (π)	Average Number of Nucleotide Differences (k)
*COI*				
	DB	0.467	0.005	3.616
	HB	0.420	0.006	3.918
	HHH	0.593	0.013	9.358
	SGN	0.265	0.001	0.720
	XB	0.419	0.001	0.419
	NF	0.543	0.003	2.324
	XN	0.712	0.002	1.238
	Total	0.526	0.006	4.237
*ITS2*				
	DB	0.171	0.000	0.192
	HB	0.559	0.007	2.870
	HHH	0.590	0.014	5.719
	SGN	0.561	0.003	1.080
	XB	0.000	0.000	0.000
	NF	0.476	0.002	0.952
	XN	0.532	0.002	0.677
	Total	0.433	0.004	1.591
*EF-1α*				
	DB	0.325	0.001	0.409
	HB	0.595	0.003	1.563
	HHH	0.643	0.005	2.691
	SGN	0.189	0.000	0.197
	XB	0.448	0.001	0.552
	NF	0.619	0.003	1.371
	XN	0.841	0.005	2.788
	Total	0.472	0.002	1.026

**Table 2:** Analysis of molecular variance (AMOVA) of *Monolepta signata* based on *COI*, *ITS2* and *EF-1α* genes.

Gene	Source of Variation	d.f.	Sum of Squares	Variance Components	Percentage of Variation
*COI*	among populations	6	298.476	0.75918 Va	30.75
	within populations	561	958.970	1.70939 Vb	69.25
	total	567	1257.445	2.46857	
*ITS2*	among populations	6	127.046	0.32550 Va	36.01
	within populations	561	324.526	0.57848 Vb	63.99
	total	567	451.572	0.90398	
*EF-1α*	among populations	6	52.872	0.13262 Va	23.97
	within populations	561	236.038	0.42075 Vb	76.03
	total	567	288.910	0.55336	

**Table 3:** Neutral test values of *Monolepta signata* based on *COI*, *ITS2* and *EF-1α* genes.

Population Code	*COI*		*ITS2*		*EF-1α*	
	Tajima’s *D* Test	Fu’s *Fs* Test	Tajima’s *D* Test	Fu’s *Fs* Test	Tajima’s *D* Test	Fu’s *Fs* Test
DB	−0.511	−1.887	−2.245 *	−26.425 *	−1.756 *	−9.543 *
HB	−0.811	0.992	−0.513	3.403	0.224	−0.741
HHH	1.914	8.077	1.497	6.584	0.878	2.111
SGN	−2.249 *	−3.556 *	−0.198	−1.882	−1.574 *	−4.741 *
XB	0.742	0.909	0.000	0.000	−0.268	−0.248
NF	0.063	2.996	1.443	2.520	0.368	0.376
XN	−1.982 *	−7.460 *	−1.628 *	−4.386 *	−0.044	−2.844
ALL	−1.412 *	−21.491 *	−1.869 *	−17.613 *	−1.681 *	−26.827 *

* *p* < 0.05.

**Table 4:** Potential distribution areas of *Monolepta signata* in different periods.

Period	Scenario	Area (×10^4^ km^2^)		
		Marginally Suitable Region	Moderately Suitable Region	Highly Suitable Region
Present	-	192.80	195.86	148.59
2041–2060	SSP126	193.03 (+0.12%)	183.54 (−6.29%)	136.48 (−8.15%)
	SSP585	218.07 (+13.11%)	165.48 (−15.51%)	126.49 (−14.87%)
2061–2080	SSP126	210.60 (+9.24%)	182.67 (−6.77%)	128.60 (−13.45%)
	SSP585	225.66 (+17.04%)	168.15 (−14.15%)	126.98 (−14.54%)

Note: The data in the brackets are the changes in area compared with current time.

## Data Availability

The data supporting the findings of this study are openly available from the National Center for Biotechnology Information at https://www.ncbi.nlm.nih.gov (accessed on 2 December 2024), accession numbers: *COI*: PQ591069, PQ609709 and PQ655538-PQ656103, *ITS2*: PQ672986-PQ673553, and *EF-1α*: PQ595069-PQ595636.

## Supplementary Materials

The following supporting information can be downloaded at: https://www.mdpi.com/article/10.3390/insects16030323/s1, Figure S1: Mismatch distribution based on data from genetic divergent clades; Figure S2: The receiver operating characteristic (ROC) curve for the optimal model parameters (a) and correlation analysis of 19 bioclimatic variables (b); Figure S3: Jackknife test of the regularized training gain for environment variables in MaxEnt; Figure S4: Response curve of main environmental variables affecting the distribution of *Monolepta signata*; Table S1: Experimental specimen information of *Monolepta signata*; Table S2: Haplotype distribution and genetic diversity of *Monolepta signata*; Table S3: Effective distribution site coordinates of *Monolepta signata*; Table S4: Climate variables and descriptions; Table S5: Genetic distance of *Monolepta signata* based on *COI* gene, *ITS2* gene, *EF-1α* genes; Table S6: The pairwise differentiation coefficient *Fst* (below the diagonal) and gene flow *Nm* (above the diagonal) of *Monolepta signata* based on *COI* gene, *ITS2* gene, and *EF-1α* genes; Table S7: Contribution rate of environmental variables to the distribution of *Monolepta signata*.
