# Supplementary material for: Population Genetics, Demographic History, and Potential Distributions of the New Important Pests Monolepta signata (Coleoptera: Chrysomelidae) on Corn in China
Source: Insects. 2025 Mar 19;16(3):323. doi: 10.3390/insects16030323 (PMC11942702; doi:10.3390/insects16030323)
Supplement: Supplementary file 1 [file insects-16-00323-s001.zip › insects-3441986-supplementary.pdf]

insects

MDPI

Supplementary Materials

Population Genetics, Demographic History, and Potential Distributions of the New Important Pests *Monolepta signata* (Coleoptera: Chrysomelidae) on Corn in China

Yang Liu [1,†], Yacong Ge [1,†], Liming Wang [1], Jingao Dong [1,*], Zhenying Wang [2,*] and Yuyu Wang [1,*]

[1] College of Plant Protection, Hebei Agricultural University, Baoding 071001, China; yangliu010204@126.com (Y.L.); ayacong@163.com (Y.G.); wanglm1990@126.com (L.W.)
[2] Institute of Plant Protection, Chinese Academy of Agricultural Sciences, Beijing 100193, China
* Correspondence: dongjingao@126.com (J.D.); wangzhenying@caas.cn (Z.W.); wangyy_amy@126.com (Y.W.)
† These authors contributed equally to this paper.

Table S1. Experimental specimen information of *Monolepta signata.*

Population name	Locality of collection	Sample code	Longitude	Latitude	Date	Sample size	Host
Dongbei population (DB)	Jiamusi, Heilongjing	JMS	130.43°	46.79°	2021-VII-17	15	corn
	Qiqihaer, Heilongjing	QQHE	123.70°	47.27°	2021-VIII-06	15	corn
	Suihua, Heilongjing	SH	126.99°	46.62°	2021-VIII-09	15	corn
	Heihe, Heilongjing	HH	127.48°	50.26°	2021-VIII-10	15	corn
	Jixi, Heilongjing	JX	131.91°	45.64°	2021-VIII-14	15	corn
	Panshi, Jilin	PS	126.07°	43.34°	2021-VII-23	15	corn
	Tonghua, Jilin	TH	126.16°	42.73°	2021-VIII-09	15	corn
	Meihekou, Jilin	MHK	125.87°	42.82°	2021-VIII-09	15	corn
	Liaoyuan, Jilin	LY	124.95°	42.97°	2021-VIII-11	15	corn
	Gongzhuling, Jilin	GZL	124.83°	43.51°	2021-VIII-10	15	corn
	Dunhua, Jilin	JLDH	128.03°	43.56°	2021-VIII-17	15	corn
	Fuxin, Liaoning	FX	121.73°	42.13°	2020-IX-17	15	corn
	Dandong, Liaoning	DD	124.05°	40.48°	2020-IX-17	15	corn
	Shenyang, Liaoning	SY	123.58°	42.04°	2021-VIII-10	15	corn
	Chaoyang, Liaoning	CY	119.80°	41.18°	2021-VIII-13	15	corn
	Dalian, Liaoning	DL	122.40°	39.81°	2021-VIII-09	15	corn
	Tieling, Liaoning	TL	123.81°	42.25°	2021-VIII-09	15	corn
	Chifeng, Neimenggu	CF	119.15°	41.87°	2021-VIII-10	15	corn
	Tongliao, Neimenggu	NMGTL	122.53°	43.78°	2021-VIII-09	15	corn
	Hulunbeier, Neimenggu	HLBE	125.40°	49.28°	2021-VIII-12	15	corn
Huabei population (HB)	Chabei Management Area, Zhangjiakou	CB	114.95°	41.41°	2020-VIII-20	15	oats
	Shalingzi, Zhangjiakou	SLZ	114.94°	40.68°	2021-VIII-08	15	corn
	Yanqing, Beijing	YQ	116.03°	40.44°	2021-VIII-18	15	corn
	Xinzhou, Shanxi	XZ	112.73°	38.46°	2021-VIII-08	15	corn
	Yuncheng, Shanxi	SXYC	110.89°	35.54	2021-VIII-10	15	corn
Huanghuaihai population (HHH)	Xianxian, Cangzhou, Hebei	XX	115.96°	38.32°	2020-IV-01	15	corn
	Hebi, Henan	HB	114.30°	35.67°	2021-VIII-17	15	corn
	Jining, Shandong	JN	116.92°	35.46°	2021-IX-07	15	corn
Shanganning population	Xian, Shanxi	XA	109.34°	34.54°	2021-VIII-11	15	corn

(SGN)							
	Xianyang, Shanxi	XY	108.41°	35.22°	2021-IX-10	15	corn
	Qingyang, Gansu	QY	107.65°	35.55°	2021-VIII-13	15	corn
	Pingliang, Gansu	PL	107.57°	35.47°	2021-IX-08	15	corn
	Guyuan, Ningxia	GY	106.29°	36.01°	2021-VIII-21	15	corn
	Eerduosi, Neimenggu	EEDS	107.28°	38.23°	2021-VIII-13	15	corn
Xibei population (XB)	Jizhang, Xinjiang	JC	87.09°	43.92°	2021-VIII-13	15	corn
Nanfang population (NF)	Yichang, Hubei	YC	111.94°	30.62°	2021-VIII-24	15	corn
Xinan population (XN)	Liangshan, Sichuan	LS	102.17°	27.62°	2021-VIII-20	15	corn
	Dehong, Yunnan	DH	98.47°	24.28°	2020-IX-24	13	corn
M. occifluvis	Beiliu, Guangxi		110.22°	22.28°	2018-IV-02		

Table S2. Haplotype distribution and genetic diversity of *Monolepta signata.*

	Population code	Haplotype
COI	DB	H_1*, H_2*, H_3, H_4, H_5*, H_6*, H_7, H_14, H_17, H_22, H_23, H_24, H_25, H_26, H_28, H_29, H_31, H_34*, H_39, H_40
	HB	H_1*, H_2*, H_5*, H_6*, H_34*, H_35, H_36, H_37, H_38, H_43*, H_47, H_48
	HHH	H_1*, H_19, H_20, H_21, H_30, H_43*, H_44
	SGN	H_1*, H_5*, H_6*, H_15, H_16, H_18, H_41, H_42
	XB	H_1*, H_27
	NF	H_9*, H_45, H_46
	XN	H_8, H_9*, H_10, H_11, H_12, H_13, H_32, H_33
ITS2	DB	H_1*, H_2*, H_5*, H_6, H_10, H_11, H_12, H_13*, H_14, H_21, H_23, H_24, H_25, H_26, H_27
	HB	H_1*, H_2*, H_3*, H_4, H_22, H_28*
	HHH	H_1*, H_2*, H_3*, H_5*, H_15, H_28*
	SGN	H_1*, H_2*, H_3*, H_5*, H_18, H_19, H_20, H_29
	XB	H_2*
	NF	H_2*, H_7*
	XN	H_2*, H_7*, H_8, H_9, H_13*, H_16, H_17
EF-1α	DB	H_1*, H_3*, H_6*, H_7, H_12*, H_13*, H_14, H_15*, H_17, H_25, H_26
	HB	H_1*, H_2, H_3*, H_4, H_5, H_6*, H_12*, H_15*
	HHH	H_1*, H_3*, H_15*, H_18, H_28, H_29
	SGN	H_1*, H_3*, H_6*, H_16, H_27, H_30
	XB	H_3*, H_6*, H_13*
	NF	H_11*, H_19*, H_22*, H_24*
	XN	H_3*, H_8, H_9, H_10, H_11*, H_19*, H_20, H_21, H_22*, H_23, H_24*

* Shared haplotypes.

Table S3. Effective distribution site coordinates of *Monolepta signata.*

Location	Longitude	Latitude
1	114.95	41.41
2	115.96	38.32
3	114.94	40.68
4	114.87	40.75
5	115.03	40.07
6	130.43	46.79

7	126.99	46.62
8	123.70	47.27
9	127.48	50.26
10	132.04	45.62
11	131.91	45.64
12	124.05	40.48
13	121.73	42.13
14	123.81	42.25
15	123.58	42.04
16	98.47	24.28
17	126.16	42.73
18	125.87	42.82
19	124.95	42.97
20	125.49	42.53
21	125.87	42.65
22	124.83	43.51
23	124.80	43.51
24	128.03	43.56
25	122.53	43.78
26	119.15	41.87
27	107.28	38.23
28	122.78	47.92
29	122.78	47.92
30	125.10	49.33
31	125.40	49.28
32	112.73	38.46
33	112.84	38.04
34	112.72	38.46
35	110.89	35.54
36	108.41	35.22
37	107.65	35.55
38	107.57	35.47
39	87.09	43.92
40	87.57	43.82
41	116.10	40.48
42	106.33	35.51
43	106.29	36.01
44	111.94	30.62
45	116.92	35.46
46	115.70	37.90
47	104.11	30.82
48	102.74	29.35
49	102.21	27.78
50	107.62	29.77
51	110.23	30.88
52	98.34	24.45
53	103.02	25.28
54	105.45	26.36
55	108.25	23.17
56	118.46	26.84
57	109.52	32.85
58	106.02	32.90
59	126.78	46.75

60	123.02	47.10
61	127.05	42.30
62	125.83	42.63
63	126.02	44.50
64	126.45	43.95
65	123.78	42.23
66	121.90	39.67
67	109.73	39.52
68	110.45	40.32
69	119.25	42.25
70	110.13	40.30
71	115.93	40.27
72	112.42	37.40
73	112.25	39.00
74	110.88	38.10
75	111.88	39.08
76	107.65	34.20
77	117.95	40.96
78	105.64	33.28
79	105.51	33.39
80	105.25	32.75
81	104.94	33.40
82	108.55	33.55
83	119.22	27.76
84	119.44	37.13
85	119.25	27.76
86	119.15	28.05
87	114.58	24.63
88	117.72	27.73
89	109.27	19.05
90	109.84	18.73
91	107.96	21.82
92	103.01	30.9
93	107.96	25.25
94	95.39	29.59
95	131.88	45.55
96	126.10	45.01
97	124.84	45.14
98	122.79	45.59
99	124.34	43.32
100	123.52	43.52
101	123.12	47.34
102	123.48	46.36
103	125.90	48.05
104	124.94	48.50
105	86.07	44.34
106	125.41	43.82
107	114.14	40.10
108	113.16	36.22
109	84.65	44.67
110	127.02	44.38
111	109.27	21.46
112	119.69	41.38

113	112.95	36.33
114	84.51	44.98
115	86.02	44.34
116	124.19	50.43
117	124.81	43.52
118	128.98	45.75
119	117.29	31.80
120	107.26	34.39
121	119.15	41.87
122	107.63	34.46
123	82.46	44.73
124	124.77	45.15
125	123.63	47.23
126	121.70	25.10
127	121.70	24.40
128	120.60	24.20
129	121.20	24.70
130	120.90	24.40
131	121.60	24.80
132	113.90	22.30
133	114.10	22.50
134	114.10	22.60
135	114.10	22.50
136	114.00	22.60
137	114.00	22.40
138	115.70	39.90
139	113.10	28.80
140	113.00	29.00
141	113.40	23.00
142	116.40	23.60
143	118.10	24.70
144	116.40	23.50

Table S4. Climate variables and descriptions.

Variable	Description
bio1	Annual mean temperature (°C)
bio2	Monthly diurnal range (°C)
bio3	Isothermality
bio4	Variation coefficient of temperature
bio5	Max temperature of the warmest month (°C)
bio6	Min temperature of the coldest month (°C)
bio7	Annual range of temperature (°C)
bio8	Mean temperature of wettest quarter (°C)
bio9	Mean temperature of the driest quarter (°C)
bio10	Mean temperature of the warmest quarter (°C)
bio11	Mean temperature of the coldest quarter (°C)
bio12	Annual precipitation (mm)
bio13	Precipitation of the wettest month (mm)
bio14	Precipitation of the driest month (mm)
bio15	Variation coefficient of precipitation
bio16	Precipitation of the wettest quarter (mm)
bio17	Precipitation of the driest quarter (mm)
bio18	Mean precipitation of the warmest quarter (mm)

bio19	Mean precipitation of the coldest quarter (mm)

Table S5. Genetic distance of *Monolepta signata* based on *COI* gene, *ITS2* gene, *EF-1α* genes.

		DB	HB	HHH	SGN	XB	NF	XN
COI	DB	0.005						
	HB	0.006	0.006					
	HHH	0.012	0.011	0.013				
	SGN	0.004	0.004	0.010	0.001			
	XB	0.004	0.004	0.010	0.001	0.001		
	NF	0.010	0.011	0.015	0.009	0.010	0.003	
	XN	0.011	0.011	0.015	0.010	0.010	0.003	0.002
ITS2	DB	0.000						
	HB	0.004	0.007					
	HHH	0.011	0.013	0.014				
	SGN	0.002	0.005	0.012	0.003			
	XB	0.000	0.004	0.010	0.002	0		
	NF	0.002	0.006	0.012	0.004	0.002	0.002	
	XN	0.005	0.009	0.016	0.007	0.005	0.004	0.002
EF-1α	DB	0.001						
	HB	0.002	0.003					
	HHH	0.004	0.005	0.005				
	SGN	0.001	0.002	0.004	0.000			
	XB	0.001	0.002	0.004	0.001	0.001		
	NF	0.003	0.004	0.006	0.003	0.003	0.003	
	XN	0.004	0.005	0.007	0.004	0.005	0.005	0.006

Table S6. Pairwise differentiation coefficient *Fst* (below the diagonal) and gene flow *Nm* (above the diagonal) of *Monolepta signata* based on *COI* gene, *ITS2* gene, *EF-1α* gene.

		DB	HB	HHH	SGN	XB	NF	XN
COI	DB	/	3.882	0.656	2.779	2.708	0.208	0.166
	HB	0.061*	/	1.511	3.627	5.842	0.212	0.154
	HHH	0.276*	0.142*	/	0.432	0.911	0.421	0.298
	SGN	0.083*	0.065*	0.367*	/	3.078	0.047	0.036
	XB	0.085*	0.041	0.215*	0.075*	/	0.065	0.040
	NF	0.546*	0.542*	0.373*	0.842*	0.794*	/	1.994
	XN	0.602*	0.619*	0.457*	0.874*	0.862*	0.111*	/
ITS2	DB	0	0.966	0.170	0.448	8.341	0.276	0.033
	HB	0.206*	/	1.410	4.147	5.975	2.022	0.292
	HHH	0.595*	0.151*	/	0.438	0.948	0.842	0.314
	SGN	0.358*	0.057*	0.363*	/	1.190	0.643	0.122
	XB	-0.029	0.040	0.209*	0.174*	/	0.625	0.062
	NF	0.4755*	0.110*	0.229*	0.280*	0.286	/	0.262
	XN	0.885*	0.461*	0.444*	0.673*	0.801*	0.489*	/
EF-1α	DB	/	1.833	0.328	33.443	3.307	0.162	0.232
	HB	0.120*	/	2.991	2.012	3.730	0.559	0.855
	HHH	0.433*	0.077*	/	0.458	1.071	0.577	0.843
	SGN	0.007	0.111*	0.353*	/	0.979	0.125	0.308
	XB	0.070*	0.063*	0.189*	0.204*	/	0.341	0.813
	NF	0.607*	0.309*	0.302*	0.667*	0.423*	/	1.280
	XN	0.518*	0.226*	0.229*	0.448*	0.235*	0.163*	/

*$p < 0.05$.

Table S7. Contribution rate of environmental variables to the distribution of *Monolepta signata*.

Variable	Contribution (%)	Permutation (%)
bio18	40.5	5.5
bio4	16.0	25.5
bio19	9.0	1.7
bio15	8.4	0.7
bio10	10.3	2.7
bio1	7.4	48.3
bio14	2.5	1.0
Bio2	4.0	0.6
Bio5	2.0	14.1

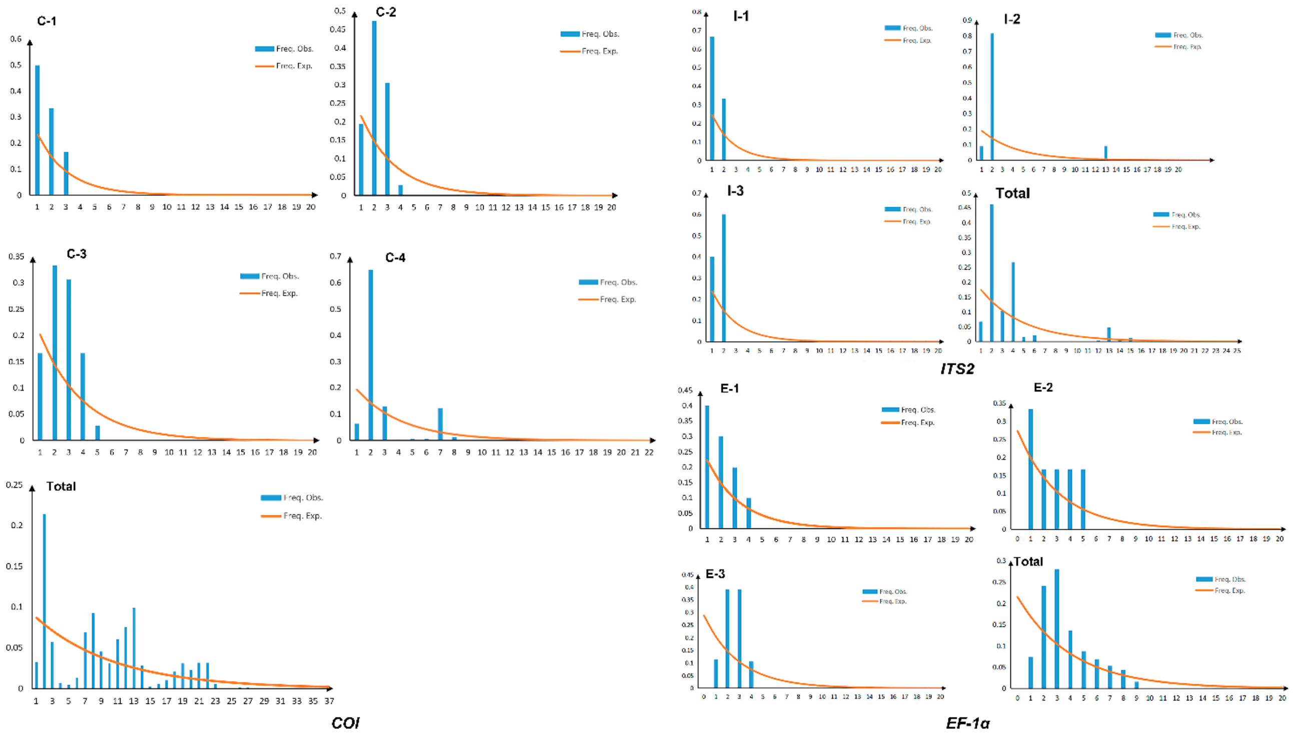

Figure S1. Mismatch distribution based on data from genetic divergent clades.

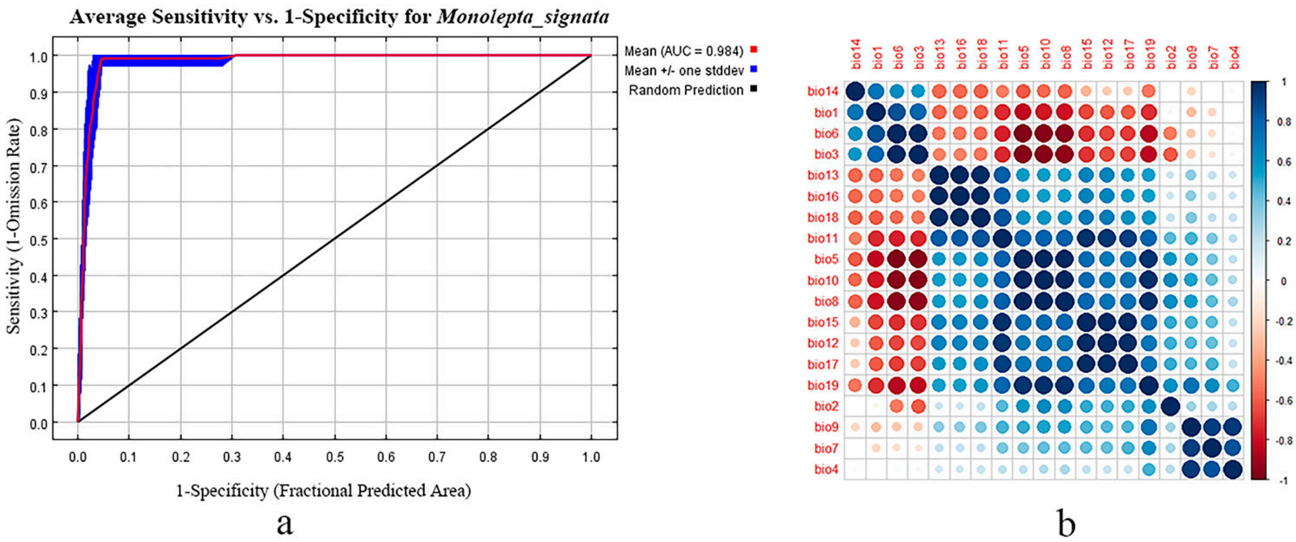

a b

Figure S2. The receiver operating characteristic (ROC) curve for the optimal model parameters (**a**) and correlation analysis of 19 bioclimatic variables (**b**).

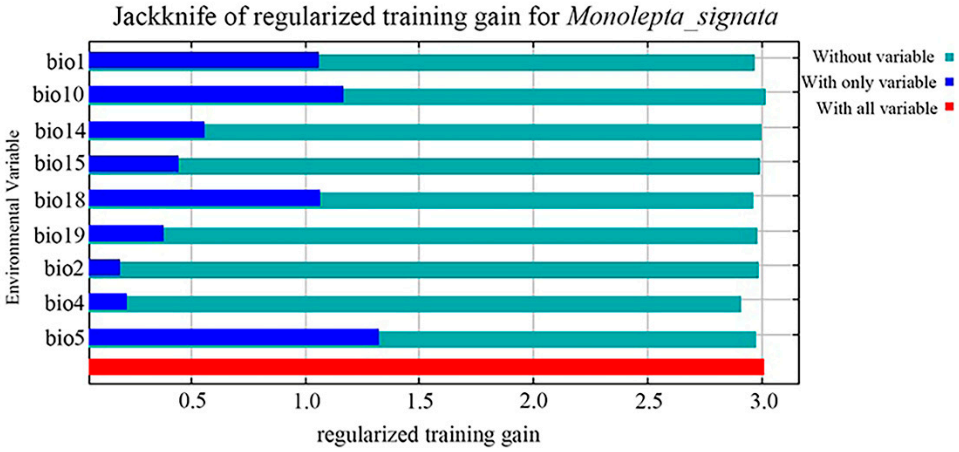

Figure S3. Jackknife test of the regularized training gain for environment variables in MaxEnt.

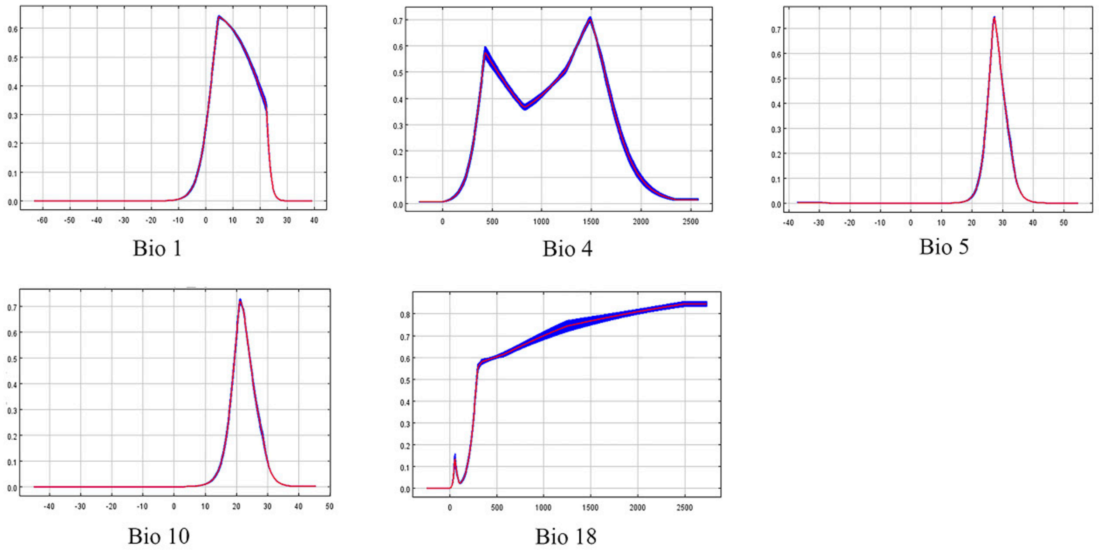

Figure S4. Response curve of main environmental variables affecting the distribution of *Monolepta signata.*
